# Microbiome-metabolomics analysis of the effects of decreasing dietary crude protein content on goat rumen mictobiota and metabolites

**DOI:** 10.5713/ab.21.0411

**Published:** 2022-03-03

**Authors:** Wen Zhu, Tianwei Liu, Jian Deng, Cong Cong Wei, Zi Jun Zhang, Di Ming Wang, Xing Yong Chen

**Affiliations:** 1 College of Animal Science and Technology, Anhui Agricultural University, Hefei 230036, China; 2 Key Laboratory of Molecular Animal Nutirtion, Ministry of Education, Zhejiang University, Hangzhou 310058, China

**Keywords:** Goat, Low Protein, Metabolomic, Microbiome, Rumen

## Abstract

**Objective:**

The objective of this study was to investigate the effects of decreasing dietary crude protein content on rumen fermentation, mictobiota, and metabolites in goats.

**Methods:**

In an 84-day feeding trial, a total of twelve male Anhui white goat kids with initial body weight 15.9±1.13 kg were selected and randomly classified into two groups, feeding a normal crude protein diet (14.8% CP, NCP) or a low crude protein diet (12.0% CP, LCP). At the end of the experimental trial (on day 84), six animals were randomly selected from each group and were slaughtered to collect rumen fluid samples for the analysis of rumen fermentation parameters, microbiome, and metabolome.

**Results:**

The concentrations of ammonia-nitrogen, total volatile fatty acid, acetate, and propionate were decreased (p<0.05) in the LCP group in comparison with those in the NCP group. The abundances of genera *Prevotella*, *Campylobacter*, *Synergistetes*, and *TG5*, which were associated with nitrogen metabolism, were lower (p<0.05) in the LCP group compared with those in the NCP group. The levels of 78 metabolites (74 decreased, 4 increased) in the rumen fluid were altered (p<0.05) by the treatment. Most of the ruminal metabolites that showed decreased levels in the LCP group were substrates for microbial protein synthesis. Metabolic pathway analysis showed that vitamin B6 metabolism was significantly different (p<0.05) in rumen fluid between the two treatments.

**Conclusion:**

Decreased dietary protein level inhibited rumen fermentation through microbiome and metabolome shifts in goat kids. These results enhance our understanding of ruminal bacteria and metabolites of goat fed a low protein diet.

## INTRODUCTION

The nitrogen (N) efficiency (the sum of N retained in milk and gain divided by N intake) of ruminants is very low (less than 30%), the rest is excreted into urine and feces [1]. The application of a low protein diet has been demonstrated to be an effective way to reduce urinary N loss and improve N utilization efficiency in ruminants [2]. However, the decreased microbial protein synthesis and nutrient digestion as a result of reduced dietary protein intake may have negative effects on goat production [3,4]. Thus, to facilitate application of a low protein diet without causing detrimental effects on production, a better understanding of the alterations that occur in response to decreasing protein content diet is needed.

The rumen is a complex microbial ecosystem that harbors a wide variety of microor ganisms, including bacteria, archaea, fungi, and protozoa [5]. The ruminal microbiota degrades and ferments dietary compounds into absorbable molecules, including volatile fatty acids (VFAs) and amino acids, which can be used by the host animal [6]. Rumen microbiota is regulated by dietary nutritional factors [7]. The protein content is one of the most important dietary nutritional factors affecting microbial growth [8]. For example, Gao et al [9] reported that the relative abundances of *Ruminobacter amylophilus* and *Ruminococcus albus* in the rumen of steer were increased with a decreasing dietary protein content. Furthermore, the rumen microbiota also coproduces a wide range of metabolites that play key roles in shuttling information between the microbial symbionts and their host [10]. Thus, a comprehensive analysis of the rumen microbe profile and metabolic processes will offer important insights for understanding the potential limitations of the application of low protein diet.

In our previous study, we found that the average daily gain and feed efficiency were decreased with a reduction in the dietary protein content [4]. We hypothesized that the phenotype differences in growing goats may be due to alterations of the rumen microbiota and metabolism induced by low dietary protein content. Therefore, this study aimed to investigate the effect of the dietary protein reduction on rumen fermentation parameters, rumen microbiota, and metabolism in growing goats.

## MATERIALS AND METHODS

### Animals and experimental design

A detailed description of the experimental design and animal treatment has been reported [4]. Briefly, animal usage was approved by the Animal Care Advisory Committee of the Anhui Agriculture University (SYXK (Wan) 2016-007). Twenty-four healthy male Anhui white goat kids (initial live weight, 15.9±1.13 kg; age, 109±4 days; n = 24) were randomly distributed into two dietary treatments. Diets differed in crude protein (CP) content as follows: i) 148 g CP/kg of dry matter (DM) (normal CP diet, NCP); ii) 120 g CP/kg of DM (low CP diet; LCP). The control diet was formulated to meet the nutrient requirements of 15 kg meat goat aimed to 100 to 150 g/d daily gain according to NRC guidelines [11] (Table 1). The feeding trial was conducted for 14 weeks, with the first 2 weeks used as an adaption period. The animals were fed twice daily at 06:30 and 18:30 h, allowing for approximately 10% feed refusal, with free access to clean water.

### Sample collection and measurement

At the end of the experiment, six goats were randomly selected from each group and were slaughtered in a local slaughterhouse before morning feeding. Homogenized rumen digesta from each goat was collected and was immediately squeezed through 4 layers of cheesecloth. The pH of the rumen fluid was measured immediately by a mobile pH meter (Seven2Go Pro S8; Mettler Toledo Co. Ltd., Shanghai, China). One subsample of each rumen filtrate sample was acidified by 25% HPO_3_ (4:1; v:v) for analysis of VFAs, as described by Hu et al [12]. Another 1.0-mL subsample was used to determine the ammonia-N content by the colorimetric method, in accordance with the method reported by Chaney and Marbach [13]. Two other 5.0-mL subsamples were infused into two 10-mL spiral centrifuge tubes for analysis of microbiota and metabolites. All collected rumen fluid samples were snap-frozen in liquid nitrogen immediately, transferred to the laboratory, and stored at −80°C for further analysis.

### Rumen microbial 16S rDNA sequencing

Rumen microbial genomic DNA was extracted using the QIAamp DNA stool mini kit (QIAGEN, Inc., Venlo, Netherlands). The integrity and concentration of the extracted DNA was evaluated using agarose gel (1%, wt/vol) electrophoresis and a NanoDrop spectrophotometer (Thermo Fisher Scientific, Waltham, MA, USA), respectively.

The amplicons of the V3-V4 regions of the 16S rRNA gene were prepared using the primers 520F/802R with dual-index 7-bp barcodes according to the method described by Wang et al [14]. After purification and quantification, amplicons for all samples were pooled in equimolar concentrations and sequenced on an Illumina MiSeq platform at Shanghai Personal Biotechnology Co., Ltd (Shanghai, China). Raw sequence data in this study are available from the National Center for Biotechnology Information Sequence Read Archive database under accession numbers SRR12597087-SRR 12597110.

### Sequence processing and statistical analyses

Raw sequences with a quality score <20, length <150 bp, and mononucleotide repeats >8 bp were filtered by QIIME pipeline (v1.8.0). Paired-end reads were merged using FLASH (1.2.8). Operational taxonomic units (OTUs) were clustered at 97% sequence similarity by UCLUST, then were classified by BLAST against the Greengenes Database (version 13.8). For alpha diversity analysis, the rarefaction and rank curves were plotted, and the Chao1 index, abundance-based coverage estimator (ACE) metric, Shannon diversity index, and Simpson index were implemented in QIIME. The beta-diversity analysis was visualized using principal-coordinate-analysis (PCoA) plots, which was estimated based on unweighted and weight UniFrac distance metrics. The analysis of similarities (ANOSIM) and permutational multivariate analysis of variance (ADONIS) were used to calculate the differences between the two groups.

### Rumen fluid metabolomics processing

Rumen samples were prepared according to the procedures described by Sun et al [15]. A total of 10 μL of each sample was mixed together as the quality control sample. A UHPLC system (1290; Agilent Technologies, Santa Clara, CA, USA) equipped with an UPLC BEH Amide column (1.7 μm×2.1 mm×100 mm; Waters, Milford, MA, USA) coupled to TOF 6600 (Q-TOF; AB Sciex, Framingham, MA, USA) was used for metabolic profiling analyses. A binary solvent system consisting of 25 mM NH_4_Ac and 25 mM NH_4_OH in water (pH 9.75) (solution A) and acetonitrile (solution B) was used with the following elution gradient procedure: 0.5 min (95% B); 6.5 min (95% to 65% B); 1 min (65% to 40% B); 1 min (40% B); 0.1 min (40% to 95% B); and 2.9 min (95% B). The column temperature and auto-sampler temperature were 25°C and 4°C, respectively. The flow rate was 0.50 mL/min, and the injection volume was 2 μL for the positive or negative mode.

The TripleTOF 6600 mass spectrometer (AB Sciex, USA) was used to obtain mass spectrometer (MS)/MS spectra with information-dependent acquisition. In each cycle, the most intensive 12 precursor ions with intensity greater than 100 were chosen for fragmentation. The collision energy (CE) was 30 eV. The cycle time was 0.56 s. The parameters of the electronic spray ionization source were as follows: 60 psi for gas 1, 60 psi for gas 2, 35 psi for the curtain gas, 600°C source temperature, 5,000 V or −4,000 ion spray voltage floating in positive or negative modes, respectively.

### Metabolomics data analysis

The MS raw data were processed by R package XCMS (version 3.22), which yielded a data matrix containing the retention time (RT), mass-to charge ratio (m/z) values, and peak intensity. Minfrac and cut-off were set as 0.5 and 0.3, respectively. The metabolites were identified using an in-house MS2 database (Biotree Biotech. Co., Ltd, Shanghai, China). A total of 3,740 resulting peaks were imported to the SIMCA software package (V15.0.2; Sartorius Stedim Data Analytics AB, Umea, Sweden) for principal component analysis (PCA) and orthogonal partial least-squares discriminant analysis (OPLS-DA). Additionally, a 7-fold permutation test with 200 random permutations in the OPLS-DA model was applied to verify the validity and robustness of the model. The metabolites were identified and confirmed by the online Kyoto encyclopedia of genes and genomes (https://www.kegg.jp/kegg/pathway.html). MetaboAnalyst 4.0 (https://www.metaboanalyst.ca/) was utilized for pathway topology analysis.

### Statistical analysis

The rumen fermentation characteristics and metabolite data were analyzed using SPSS (version 23.0; SPSS Inc., Chicago, IL, USA). One-way analysis of variance was performed to analyze the differences in pH, ammonia-N, and VFAs between the two groups. A significant change was observed at p<0.05.

The Metastats test in Mothur (version 1.30.1) was used to identify rumen bacteria that showed different abundances between the groups. Significance was observed at p<0.05 and a risk of false discovery (q value) <0.10. The value of variable importance in projection (VIP) was obtained from the OPLS-DA model, and variables with VIP>1 and p<0.05 (student’s t-test) were considered to indicate significant differences in the levels of metabolites.

## RESULTS

### Rumen fermentation parameters

Rumen pH was not significantly different (p>0.05) between the LCP and the NCP groups (Table 2). The rumen fluid concentrations of ammonia-N, total VFA, acetate, and propionate were significantly lower (p<0.05) in the LCP group in comparison with those in the NCP group. The concentrations of butyrate, valerate, and acetate to propionate ratio were not significantly altered by changes in the dietary CP level (p>0.05).

### Rumen fluid microbial communities

After quality filtering, a total of 388,089 high-quality sequences were obtained with an average of 32,341±58 (mean±standard deviation [SD]) sequences per sample, and 1,618±299 (mean ±SD) OTUs were observed based on 97% similarity. Good’s coverage for all rumen fluid samples were greater than 99.5%. The flattened rarefaction (Supplementary Figure S1A) and rank abundance curves (Supplementary Figure S1B) demonstrated that most of the rumen microbes in the samples were quantified, and the data could be used for further analysis. Alpha diversity analysis indicated that neither the richness estimators (Chao 1 and ACE value) nor the diversity indices (Shannon and Simpson indices) were affected (p>0.05) by the LCP (Table 3).

**Figure 1:**
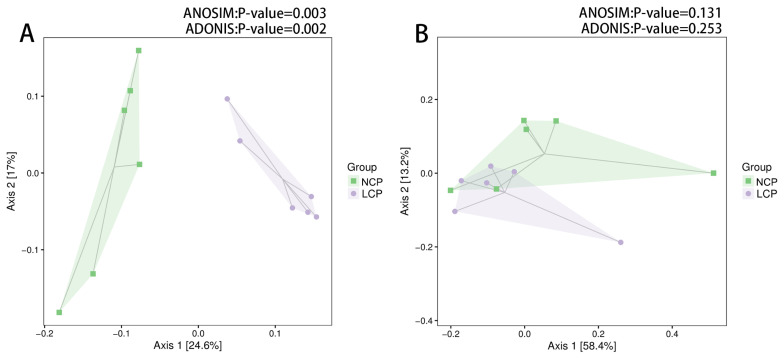
Principal coordinate analysis of unweighted (A) and weight (B) UniFrac distances of all 12 rumen samples. NCP, normal crude protein diet; LCP, low crude protein diet. ANOSIM, analysis of similarities; ADONIS, permutational multivariate analysis of variance.

Weighted Unifrac distances did not show complete separation between the two groups (Figure 1B). However, the PCoA based on the unweighted UniFrac distances indicated rumen fluid samples from the LCP and NCP groups clustered differently (ANOSIM, p<0.05; ADONIS, p<0.05) (Figure 1A).

**Figure 2:**
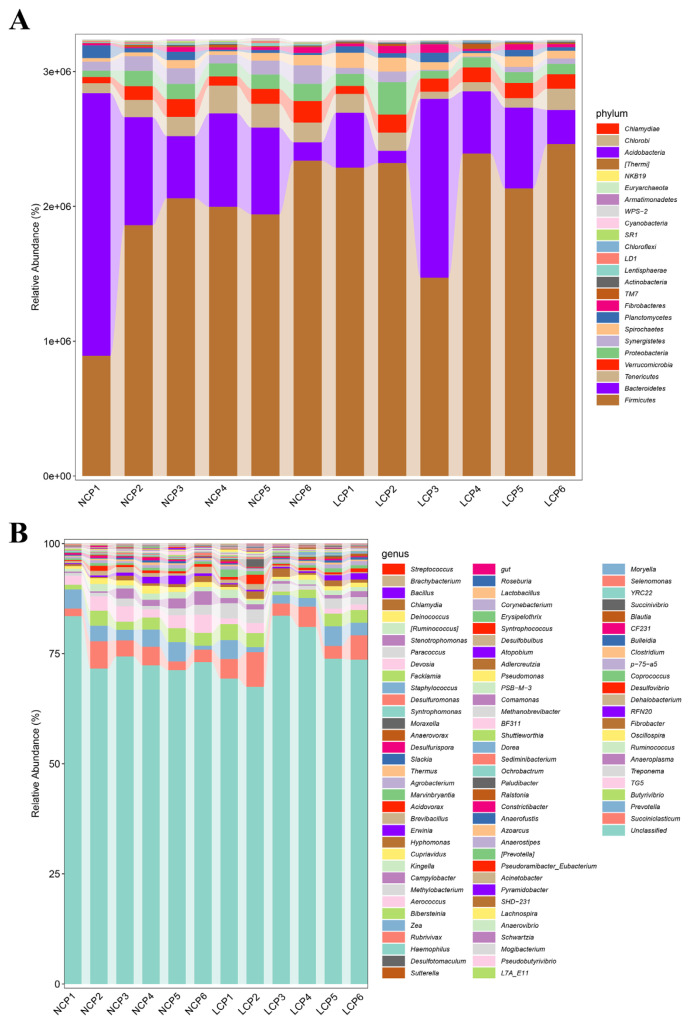
The relative abundance of rumen microbiota at the phylum (A) and genus level (B). NCP, normal crude protein diet; LCP, low crude protein diet.

The rumen-microbiota distributions at the phylum and genus levels are shown in Figure 2. Among the identified phyla, Firmicutes was the most dominant, with an average abundance of 62.3%±13.9% (mean±SD), followed by Bacteroidetes (20.2%±16.2%), Tenericutes (3.85%±1.49%), Verrucomicrobia (3.20%±1.00%), and Proteobacteria (3.15% ±1.54%) (Figure 2A). At the genus level, *Succiniclasticum* (4.06%±1.81%), *Prevotella* (3.00%±1.32%), *Butyrivibrio* (2.53%±0.91%), TG5 (1.99%±1.30%), and *Treponema* (1.73% ±0.93%) were considered as high-abundance (Figure 2B).

**Figure 3:**
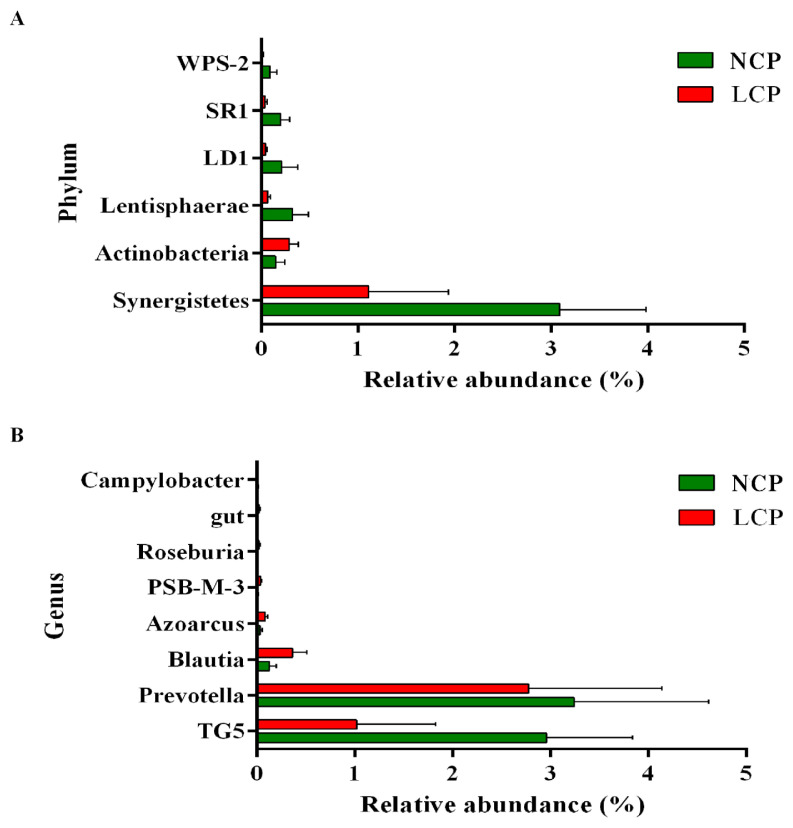
Different composition of rumen microbiota at the phylum (A) and genus (B) levels between the low crude protein diet (LCP) and the normal crude protein diet (NCP) (Metastats test).

The bacteria that were significantly different between the two groups at the phylum and genus levels are shown in Figure 3. At the phylum level (Figure 3A), in comparison with the NCP group, the LCP group showed lower (p<0.05) relative abundance of Synergistetes, Lentisphaerae, LD1, SR1, WPS-2, whereas Actinobacteria showed higher relative abundances (p<0.05). At the genus level (Figure 3B), the LCP group showed lower (p<0.05) relative abundances of *Prevotella*, TG5, and *Campylobacter* and higher (p<0.05) relative abundances of *Blautia*, *Azoarcus*, *PSB-M-3*, and *Roseburia*.

### Rumen fluid metabolic profiles

**Figure 4:**
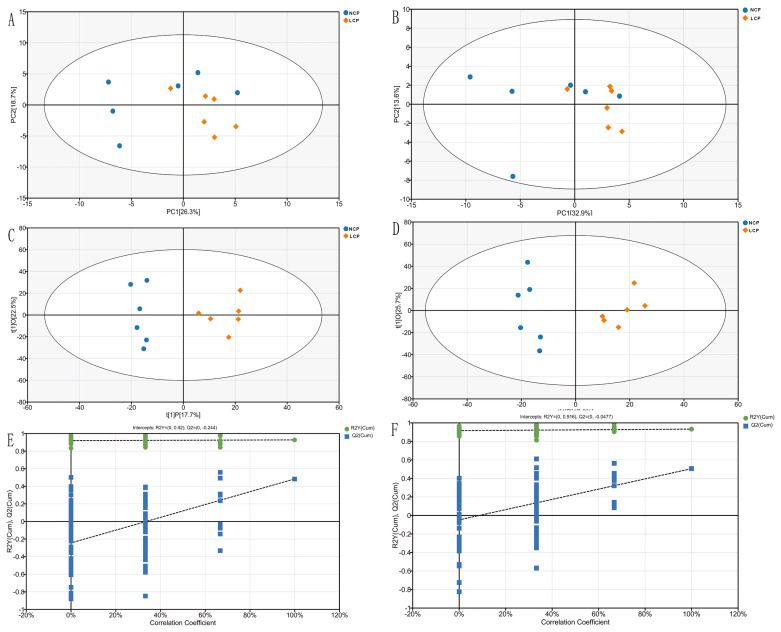
Identification of discriminating biomarkers by metabolomics analysis between the low crude protein diet (LCP) and the normal crude protein diet (NCP). The principal component analysis (A) and orthogonal partial least-squares discriminant analysis (C) score plot are in negative ion mode. The principal component analysis (B) and orthogonal partial least-squares discriminant analysis (D) score plot are in positive ion mode. Permutation tests conducted with 200 random permutations in the orthogonal partial least-squares discriminant analysis model is built for negative (E) and positive ion mode (F).

Based on the analysis of untargeted metabolomics data, PCA plots were not completely separated between the LCP and NCP groups (Figure 4A, B). However, the analysis of OPLS-DA (R^2^Y = 0.877, Q^2^ = 0.437 for the positive ion mode; R^2^Y = 0.859, Q^2^ = 0.455 for the negative ion mode) of the metabolic profiles showed a noticeable separated cluster between the LCP and NCP groups (Figure 4C, D). The corresponding Q^2^ values of the OPLS-DA model are negative (−0.58 and −0.62 in positive and negative ion mode, respectively) (Figure 4E, F), indicated the valid and at low risk of over fitting of the statistical models. In addition, in PCA and OPLS-DA plots we found all samples of the two groups within the 95% Hotelling’s T-Squared Ellipse.

The levels of 78 metabolites in the rumen fluid differed significantly (p<0.05) between the LCP and NCP groups (p<0.05 and VIP>1) (47 in the positive-ion mode and 31 in the negative-ion mode). Among these 78 metabolites, the levels of 74 were lower and 4 were higher in the LCP group in comparison with the NCP group (Supplementary Table S1). Based on the classification information, the differential metabolites were mainly classified into lipids and lipid-like molecules, phenylpropanoids and polyketides, organoheterocyclic compounds, organic oxygen compounds, benzenoids, alkaloids and derivatives, and organic acids and derivatives.

**Figure 5:**
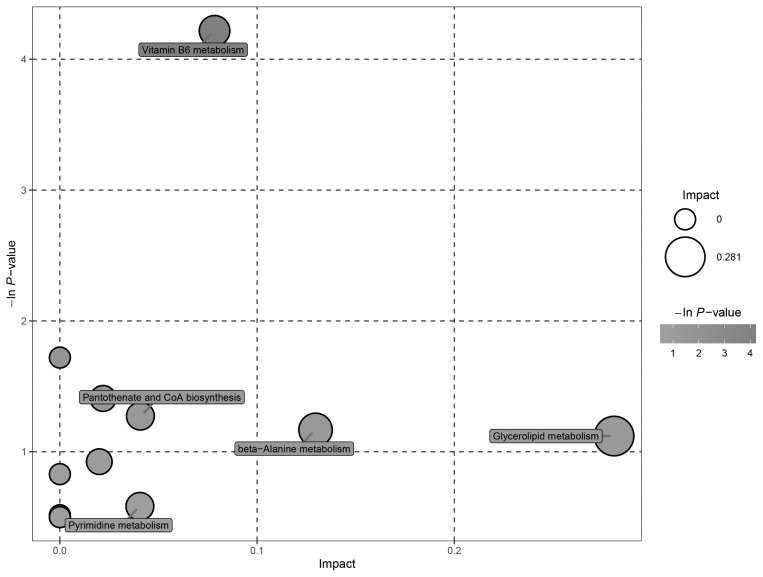
Ruminal metabolomics pathway analysis of goats that received the low crude protein diet compared with the normal crude protein diet. The sizes of the shapes represent the effects of low protein treatment on rumen fluid metabolism relative to the control treatments; larger shapes indicate greater effects on the pathway.

Data from the negative and positive modes were combined. The significantly altered metabolites between the LCP and NCP groups were enriched in pathways of vitamin B_6_ metabolism, glycerolipid metabolism, pantothenate and CoA biosynthesis, beta-Alanine metabolism, and pyrimidine metabolism (Figure 5). The detailed results of the pathway analyses are shown in Table 4. The pathway of vitamin B_6_ metabolism was significantly altered (p<0.05) by the low protein diet.

## DISCUSSION

Dietary CP level is a vital factor in the growth of ruminants. Decreasing CP level decreased the CP intake and growth performance of goat kids [4]. Ammonia-N is the final product of the feed protein decomposition, and a positive correlation between CP intake and ammonia-N has been reported by Xia et al [16]. In the present study, low protein diet decreased ammonia-N concentration, which is consistent with the previous study [17]. The VFAs, which are the primary energy source for ruminants, are the main fermentation product in the rumen [18]. The decreased concentration of total VFA, acetate, and propionate with LCP indicated less energy supply to goats, which provided a potential explanation for the decreased growth performance as was observed in our previous study [4].

Rumen microbiota composition shifts in response to dietary CP level has been reported in previous study [16]. In the current study, specific microbes were observed to differ between the two groups. At the genus level, the relative abundances of *Prevotella*, TG5, and *Campylobacter* were lower in the LCP group than in the NCP group. *Prevotella* has been reported to be involved in the degradation of oligopeptides into amino acids, which is a limiting step of rumen proteolysis [19]. *Campylobacter* is a microaerophilic bacterium that could consume oxygen and is involved in nitrogen metabolism [20]. In the rumen, *Synergistetes* is known for its capability to degrade amino acids [21]. The decreased relative abundance of *Synergistetes* in the rumen of goat fed a low protein diet was also reported by Zhang et al [22]. The decreased apparent digestibility of CP in the LCP group observed in our previous study [4] could be partially explained by the reduced abundances of these protein-degrading bacteria. Additionally, *Prevotella* have the ability to degrade fiber, partially explaining the lower acetate concentrations in the low-protein groups.

The metabolites in rumen fluid mainly contain nutrients that can be used by the host, and differences in the levels of ruminal metabolites are known to be associated with changes in microbiota [23]. In this study, most of the altered metabolites were decreased by low protein diet, including amino acids, peptides, and fatty acids. Amino acids and peptides can serve as precursors for the synthesis of microbial protein in the rumen, while fatty acids can provide energy for the synthesis of microbial proteins [18]. Thus, these results suggest that feeding a low protein diet to goats might decrease the microbial protein yield. The positive effect of dietary CP level on microbial protein synthesis is also reported by Chanthakhoun et al [24]. Glycerol was one of the significantly decreased metabolites in the LCP group in comparison with the NCP group. In the rumen, glycerol is completely fermented into VFAs, especially propionate and butyrate [25]. The decreased concentration of propionate in LCP group may be partially attributed to decreased glycerol levels.

The pathway of vitamin B _6_ metabolism was significantly altered by the dietary protein content, which was characterized by decreased concentrations of pyridoxine and 4-Pyridoxic acid. Pyridoxine is one of the vitamin B_6_ vitamers [26]. 4-Pyridoxic acid is the major urinary catabolite of vitamin B_6_ [27]. The decreased levels of pyridoxine and 4-pyridoxic acid in the LCP group indicated downregulation of vitamin B_6_ metabolism. It is well known that vitamin B_6_, mainly synthesized in rumen, as a co-factor for more than 150 enzymes, participates in carbohydrate, protein, and lipid metabolism [28]. Meale et al [29] also reported that the efficiency of blood vitamin B_6_ metabolism was reduced in low feed conversion efficient bulls. Overall, the results indicating that the reduced feed efficiency in LCP group observed in our previous study might be due to the reduced efficiency of this pathway.

## CONCLUSION

In conclusion, a decreased dietary protein content altered rumen microbial composition and rumen metabolism. Decreasing the protein content inhibited rumen fermentation, decreased the relative abundances of genera *Prevotella*, *Campylobacter*, *Synergistetes*, and TG5. Most of the metabolites that showed decreased levels in the rumen fluid in the LCP group were substrates for microbial protein synthesis, indicating a lower microbial protein yield when goats were fed LCP. The pathway enrichment analysis of the different metabolites indicated that LCP mainly affected vitamin B_6_ metabolism. These results enhance our understanding of ruminal bacteria and metabolites of different protein content within goat diet, and provide more informantion for the development of low protein diet.

## Figures and Tables

**Table 1:** Ingredients and chemical composition of the experimental diets^1)^

Item	Dietary treatments, % dry matter	

	NCP	LCP
Ingredient		
Ground corn grain	13.0	21.0
Soybean meal^2)^, 43.5% CP	24.0	16.0
Wheat bran	7.5	7.5
Sodium bicarbonate	1.0	1.0
Salt	1.0	1.0
Dicalcium phosphate	0.5	0.5
Calcium carbonate	1.0	1.0
Premix^3)^	1.0	1.0
Peanut vine	28.0	28.0
Chinese wild rye	22.0	22.0
Chemical composition		
Organic matter	86.3	88.3
Crude protein	14.8	12.0
Neutral detergent fiber	46.4	44.8
Acid detergent fiber	31.8	31.2
Ether extract	3.74	3.27
Digestible energy^4)^ (MJ/kg)	13.9	13.8

NCP, normal crude protein diet; LCP, low crude protein diet.

1) Cited from Zhu et al [4].

2) Soybean meal contained: 89.5% dry matter, 43.5% crude protein, 28.2% neutral detergent fiber and 10.5% acid detergent fiber.

3) Formulated to provide (per kg of dry matter): 600,000 IU of vitamin A, 80,000 IU of vitamin D_3_, 5,000 IU of vitamin E, 8,000 mg of Zn, 60 mg of Se, 200 mg of I, 9,400 mg of Fe, 72 mg of Co, 10,400 mg of Mn, and 1,600 mg of Cu; Peanut vine contained: 91.3% dry matter, 7.17% crude protein, 57.2% neutral detergent fiber, and 50.5% acid detergent fiber on dry matter basis; Chinese wild rye contained: 89.6% dry matter, 6.7% crude protein, 67.5% neutral detergent fiber, and 38.2% acid detergent fiber on dry matter basis;

4) Calculated according to nutrient requirements of small ruminants.

**Table 2:** Effects of low crude protein diet on rumen fermentation characteristics in fattening goats (n = 6)

Items	Treatment		SEM	p-value

	NCP	LCP		
pH	6.64	6.51	0.038	0.49
Ammonia-nitrogen (mg/dL)	10.5^a^	8.94^b^	0.431	0.03
Total volatile fatty acid (mM)	74.3^a^	65.1^b^	2.51	0.03
Acetate (mM)	52.6^a^	45.8^b^	1.71	0.04
Propionate (mM)	13.1^a^	11.2^b^	0.46	0.02
Butyrate (mM)	7.78	7.28	0.37	0.59
Valerate (mM)	0.81	0.83	0.029	0.67
Acetate:propionate	3.73	3.75	0.121	0.68

NCP, normal crude protein diet; LCP, low crude protein diet; SEM, standard error mean.

a,b Values within a row with different superscripts differ significantly at p<0.05. The p-values were determined using one-way analysis of variance.

**Table 3:** Alpha diversity of rumen bacterial communities in fattening goats fed the low crude protein diet (LCP) and the normal crude protein diet (NCP)

Items	Treatment		SEM	p-value

	NCP	LCP		
Good’s coverage	0.995	0.995	0.0069	0.99
Chao1 value	1,594	1,677	50.1	0.43
ACE value	1,607	1,665	48.9	0.57
Shannon indices	8.59	8.62	0.127	0.91
Simpson indices	0.991	0.992	0.0161	0.84

NCP, normal crude protein diet; LCP, low crude protein diet; SEM, standard error mean; ACE, abundance-based coverage estimator.

The p-values were determined using student’s t test.

**Table 4:** Rumen fluid metabolomic pathway analyses in fattening goat fed the low crude protein diet (LCP) and the normal crude protein diet (NCP)

Pathway	Total	Hits^1)^	p-value^2)^	Impact^3)^	Hits conpounds
Vitamin B_6_ metabolism	9	1	0.015	0.000	4-Pyridoxic acid
Arginine and proline metabolism	44	1	0.245	0.022	Argininosuccinic acid
Glycerolipid metabolism	18	1	0.326	0.281	Glycerol
Alanine, aspartate and glutamate metabolism	23	1	0.397	0.020	Argininosuccinic acid
Galactose metabolism	26	1	0.436	0.000	Glycerol
Tryptophan metabolism	41	1	0.596	0.000	Indoleacetic acid

1) Hits represent the number of metabolites matches in one pathway.

2) The p-values were determined using student’s t test.

3) Impact is the influencing factor of the pathway obtained by topology analysis.
