# Supplementary material for: Microbiome-metabolomics analysis of the effects of decreasing dietary crude protein content on goat rumen mictobiota and metabolites
Source: Anim Biosci. 2022 Mar 3;35(10):1535–44. doi: 10.5713/ab.21.0411 (PMC9449381; doi:10.5713/ab.21.0411)
Supplement: Supplementary file 1 [file ab-21-0411-suppl1.pdf]

Table S1. The list of ruminal fluid metabolites significantly differences between the low crude protein diet and the normal crude protein diet

Metabolites	Score	MZ	Retention time (s)	Ion mode	VIP	p-value[1)]	Fold Change[2)]
Yohimbine	0.332	335.18	42.23	NEG	1.878	0.00	0.650
Vincamine	0.495	355.20	374.18	POS	1.616	0.04	0.719
Phenylbutazone	0.335	307.15	42.20	NEG	1.734	0.02	0.803
Tamsulosin	0.404	407.17	322.07	NEG	2.016	0.01	0.801
3-Phenylpropanoic acid	0.879	149.06	94.20	NEG	2.271	0.00	0.693
Vanylglycol	0.420	165.05	305.52	NEG	1.902	0.02	0.605
Gemfibrozil	0.500	249.15	46.23	NEG	1.803	0.01	0.589
Pyrocatechol	0.478	152.07	117.62	POS	1.849	0.05	0.813
Phentermine	0.352	337.20	41.22	POS	1.682	0.04	0.761
12-Oxo-2,3-dinor-10,15-phytodienoic acid	0.362	301.12	61.87	NEG	1.561	0.05	0.844
20-hydroxy-PGF2a	0.610	369.22	140.14	NEG	1.822	0.03	0.833
Hexadecanedioic acid	0.484	345.23	139.01	NEG	2.040	0.02	0.826
Pimelic acid	0.872	159.07	328.85	NEG	1.871	0.02	0.824
Azelaic acid	0.986	187.10	306.54	NEG	1.870	0.02	0.811
Suberic acid	0.905	173.08	319.56	NEG	2.164	0.01	0.783
Pindone	0.450	229.09	157.72	NEG	2.000	0.01	0.771
Adipic acid	0.878	141.02	419.64	NEG	1.783	0.04	0.762
Sebacic acid	0.977	201.11	292.01	NEG	2.042	0.01	0.754
5-Hydroxyhexanoic acid	0.881	131.07	210.91	NEG	2.019	0.00	0.657
Caprylic acid	0.448	159.10	154.75	NEG	2.107	0.00	0.630
Stearoylcarnitine	0.983	428.37	148.99	POS	1.841	0.03	1.991

Glycerol 1-myristate	0.730	285.24	44.46	POS	1.888	0.01	1.459
Exemestane	0.303	373.09	261.28	POS	2.092	0.01	0.801
Citramalic acid	0.386	148.04	39.52	POS	1.352	0.04	0.757
Stearidonic Acid	0.845	277.22	86.09	POS	2.105	0.01	0.703
1-Palmitoyl-2-linoleoyl-sn-glycero-3-phosphate	0.958	671.46	150.49	NEG	1.625	0.05	0.732
6k-PGF1alpha-d4	0.809	373.26	275.40	NEG	1.961	0.01	0.574
1-Stearoyl-sn-glycerol	0.820	379.28	90.70	NEG	1.747	0.02	1.157
DL-Homocysteine	0.467	153.07	314.78	POS	1.830	0.04	0.894
Arg-Met	0.381	270.13	368.28	POS	1.874	0.03	0.873
Met-Ser	0.378	300.10	265.98	POS	1.801	0.03	0.829
Vigabatrin	0.473	171.11	347.85	POS	1.610	0.02	0.822
His-Val	0.372	318.16	314.77	POS	2.363	0.00	0.801
gamma-L-Glu-epsilon-L-Lys	0.411	339.16	339.96	POS	1.947	0.01	0.788
Trans-4-Hydroxy-L-proline	0.621	96.04	49.99	POS	1.850	0.04	0.784
DL-a-Hydroxybutyric acid	0.457	168.07	42.83	POS	1.886	0.02	0.772
Scopoline	0.633	156.10	48.97	POS	1.652	0.02	0.752
N2-Acetyl-L-ornithine	0.500	349.20	41.22	POS	2.319	0.00	0.742
Phenylacetylglycine	0.498	176.07	301.79	POS	2.145	0.01	0.730
N-Acetyl-L-tyrosine	0.446	224.09	274.40	POS	1.952	0.02	0.710
D-Alanyl-D-alanine (D-Ala-D-Ala)	0.499	143.08	135.97	POS	1.937	0.02	0.702
Hexanoylglycine	0.359	347.22	28.49	POS	1.872	0.01	0.695
Argininosuccinic acid	0.303	313.11	226.92	POS	1.622	0.02	0.682
N1-Acetylspermine	0.361	305.25	201.08	POS	1.929	0.01	0.673
alpha-Ketocaproic acid	0.417	259.12	322.32	NEG	1.405	0.05	0.687

3-Methylthiopropionate	0.453	162.06	309.35	POS	1.812	0.03	0.815
His-Ser	0.496	243.11	330.86	POS	2.265	0.00	0.755
His-Thr	0.302	256.12	287.22	POS	1.709	0.03	0.753
Pro-Trp	0.497	284.13	323.48	POS	1.637	0.02	0.746
Val-Phe	0.402	325.18	398.44	POS	1.865	0.02	0.738
Thr-Ser	0.617	248.12	363.96	POS	2.083	0.01	0.720
Ala-Lys	0.914	218.15	444.30	POS	1.810	0.02	0.714
Lys-Gly	0.626	221.17	222.42	POS	1.874	0.04	0.418
L-Sorbose	0.311	161.04	348.65	NEG	1.827	0.01	0.768
D-Mannitol 1-phosphate	0.616	321.17	41.57	NEG	2.015	0.03	0.694
3-Hexanone	0.500	121.07	47.66	NEG	1.762	0.03	0.560
L-Threonate	0.446	136.03	26.12	POS	1.866	0.03	1.068
Glycerol	0.698	171.01	379.58	NEG	2.072	0.01	0.812
Allantoin	0.444	158.04	79.43	NEG	1.736	0.04	0.824
Temazepam	0.316	316.09	294.27	NEG	1.911	0.05	0.702
Saccharin	0.339	183.00	65.36	NEG	2.070	0.00	0.656
Ethosuximide	0.378	162.05	46.15	NEG	1.943	0.04	0.516
Pyridostigmine cation	0.473	245.11	373.43	POS	1.657	0.04	0.865
5-Methoxyindoleacetate	0.499	266.10	247.62	POS	1.873	0.04	0.812
Benomyl	0.369	290.14	377.48	POS	1.710	0.03	0.797
Donepezil	0.455	312.15	224.44	POS	2.031	0.01	0.749
4-Pyridoxic acid	0.996	184.06	39.52	POS	1.575	0.02	0.722
Oxindole	0.964	134.06	40.18	POS	1.664	0.04	0.679
Indoleacetic acid	0.915	176.07	55.57	POS	2.209	0.01	0.739

Clomipramine	0.480	260.10	266.10	POS	1.704	0.04	0.826
Pyridoxine	0.995	170.08	117.85	POS	1.711	0.03	0.663
DL-3-Phenyllactic acid	0.652	165.05	46.03	NEG	1.948	0.01	0.722
Equol	0.316	207.08	140.01	POS	1.640	0.04	0.857
2,5-Dihydroxycinnamic acid methyl ester	0.394	236.09	214.37	POS	2.063	0.01	0.779
norpropoxyphene	0.320	290.20	280.32	POS	1.788	0.02	0.778
Coumarin	0.401	369.39	24.60	POS	1.940	0.04	0.575
trans-cinnamate	0.384	209.08	128.29	POS	2.150	0.01	0.829
Adynerin	0.797	515.30	119.27	NEG	1.847	0.02	0.870

MZ, mass-to-charge ratio; NEG, negative; POS, positive; VIP, variable importance in projection

[1)]p -values were calculated according to Student's T-test (n=6).

[2)] If the fold change value is less than 1, it means that there is less metabolite in the low crude protein group than in the normal crude protein group.
