# Supplementary material for: Microbiome-metabolomics analysis of the effects of decreasing dietary crude protein content on goat rumen mictobiota and metabolites
Source: Anim Biosci. 2022 Mar 3;35(10):1535–44. doi: 10.5713/ab.21.0411 (PMC9449381; doi:10.5713/ab.21.0411)
Supplement: Supplementary file 2 [file ab-21-0411-suppl2.pdf]

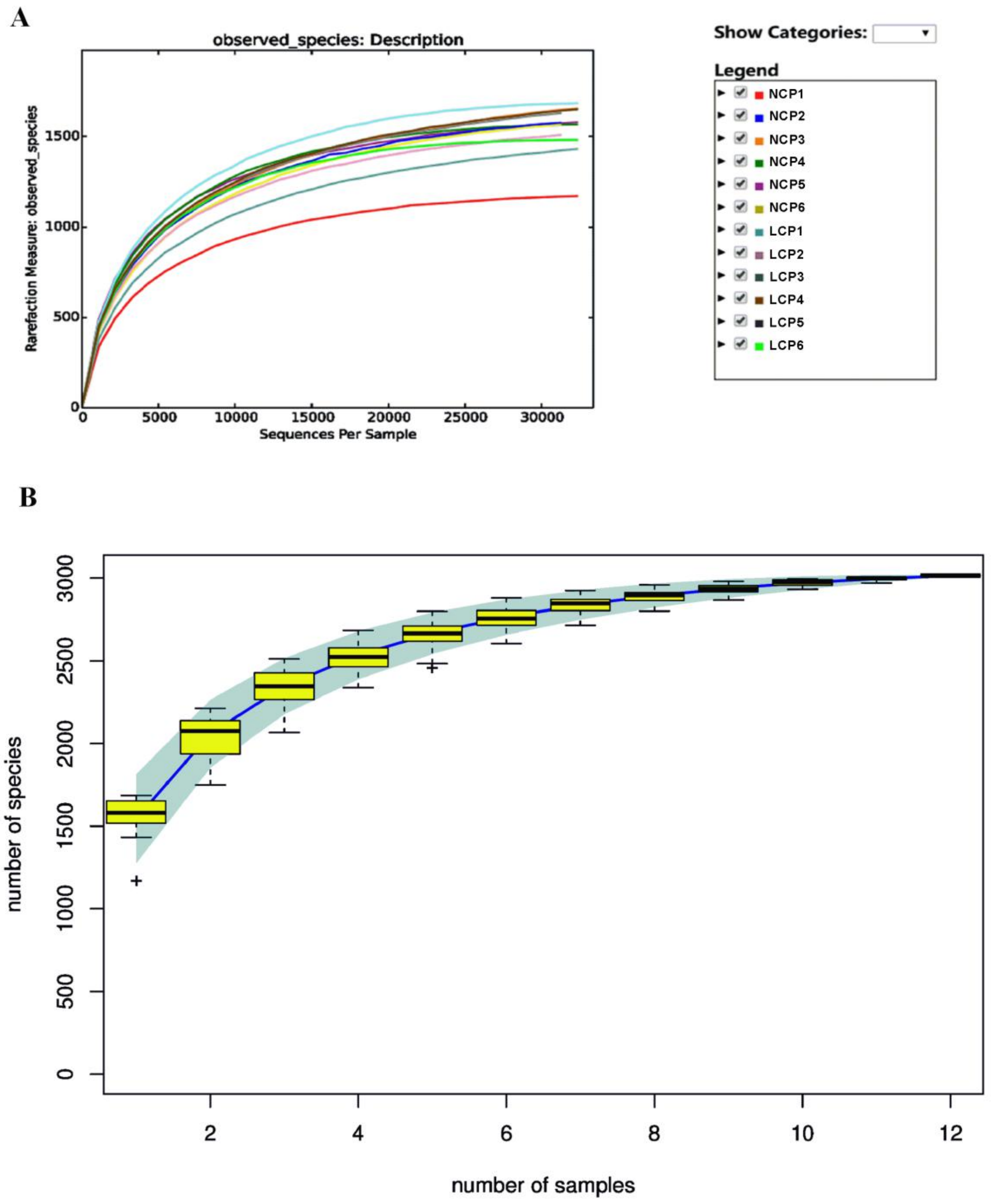

Figure S1. The flattened rarefaction (A) and rank abundance curves (B) obtained based on assigned operational taxonomic units of rumen bacteria of goats fed the low crude protein diet (LCP) and the normal crude protein diet (NCP).

.
